# Role of Cyclooxygenase-2 on Intermittent Hypoxia-Induced Lung Tumor Malignancy in a Mouse Model of Sleep Apnea

**DOI:** 10.1038/srep44693

**Published:** 2017-03-16

**Authors:** Noelia Campillo, Marta Torres, Antoni Vilaseca, Paula Naomi Nonaka, David Gozal, Jordi Roca-Ferrer, César Picado, Josep Maria Montserrat, Ramon Farré, Daniel Navajas, Isaac Almendros

**Affiliations:** 1 Unitat de Biofísica i Bioenginyeria, Facultat de Medicina, Universitat de Barcelona, 08036-Barcelona, Spain; 2 Institute for Bioengineering of Catalonia, 08028-Barcelona, Spain; 3 Centro de Investigación Biomédica en Red de Enfermedades Respiratorias, 28029-Madrid, Spain; 4 Laboratori del Son. Servei de Pneumologia, Hospital Clinic, Universitat de Barcelona 08036-Barcelona, Spain; 5 Department of Pediatrics, Pritzker School of Medicine, Biological Sciences Division, The University of Chicago, Chicago, IL 60637, USA; 6 Institut d’Investigacions Biomèdiques August Pi i Sunyer, 08036-Barcelona, Spain; 7 Department of Pneumology and Respiratory Allergy, Hospital Clinic, 08036-Barcelona, Spain

## Abstract

An adverse role for obstructive sleep apnea (OSA) in cancer epidemiology and outcomes has recently emerged from clinical and animal studies. In animals, intermittent hypoxia (IH) mimicking OSA promotes tumor malignancy both directly and via host immune alterations. We hypothesized that IH could potentiate cancer aggressiveness through activation of the cyclooxygenase-2 (COX-2) pathway and the concomitant increases in prostaglandin E2 (PGE_2_). The contribution of the COX-2 in IH-induced enhanced tumor malignancy was assessed using celecoxib as a COX-2 specific inhibitor in a murine model of OSA bearing Lewis lung carcinoma (LLC1) tumors. Exposures to IH accelerated tumor progression with a tumor associated macrophages (TAMs) shift towards a pro-tumoral M2 phenotype. Treatment with celecoxib prevented IH-induced adverse tumor outcomes by inhibiting IH-induced M2 polarization of TAMs. Furthermore, TAMs isolated from IH-exposed mice treated with celecoxib reduced the proliferation of LLC1 naïve cells, while the opposite occurred with placebo-treated IH-exposed mice. Finally, *in vitro* IH exposures of murine macrophages and LLC1 cells showed that both cell types increased PGE_2_ release in response to IH. These results suggest a crucial role for the COX-2 signaling pathway in the IH-exacerbated malignant processes, and designate macrophages and lung adenocarcinoma cells, as potential sources of PGE_2_.

Obstructive sleep apnea (OSA) is a prevalent condition characterized by repetitive occlusions of the upper airway during sleep resulting in intermittent hypoxia (IH), increased respiratory efforts and sleep fragmentation^1^. Over the last several decades, epidemiological, clinical and experimental evidences have provided significant support to the contention that OSA, and more specifically IH, operates as an independent risk factor for cardiovascular, cognitive and metabolic morbidities^2^,^3^,^4^. More recently, a potential link between OSA and cancer incidence and mortality has also been proposed based on epidemiological and animal studies^5^,^6^,^7^,^8^,^9^.

The presence of hypoxia within the tumor microenvironment can impair anti-cancer immunity responses through alterations in the secretion of cytokines, chemokines and other pro-inflammatory molecules that mediate the cross-talk between tumor cells and immune cells in the tumor stroma^10^,^11^,^12^. In the context of OSA, recent studies indicate that IH can facilitate cancer progression mainly through alterations in the host immune response^13^,^14^,^15^. More specifically, IH increases the mobilization of tumor-associated macrophages (TAMs) into the tumor, and accelerates their transformation from an anti-tumoral phenotype (M1) to a tumor-promoting phenotype (M2). In addition to TAMs, the recruitment of other immunosuppresive populations such as myeloid-derived suppressor cells (MDSCs) or regulatory T lymphocytes (Tregs) from surrounding tissues is also enhanced by IH^13^,^14^,^15^. Taken together, the IH-induced changes on the tumoral immune response would be anticipated to lead to a more tumor-permissive microenvironment, thus facilitating processes such as tumor growth, invasion, or dissemination, as previously reported^8^,^9^,^13^.

The cyclooxygenase-2 (COX-2) downstream metabolite prostaglandin E_2_ (PGE_2_) has been proposed to play a critical role in the interplay between tumor cells and stromal cells^16^,^17^. PGE_2_ can promote tumor cell proliferation, migration and invasion either directly, or indirectly through induction of a tumor-permissive microenvironment characterized by impaired immunosurveillance and increased angiogenesis^16^,^17^,^18^. Indeed, several reports have shown that COX-2 is up-regulated in different types of tumors, including colorectal, breast or lung carcinomas, and such altered expression of COX-2 is directly correlated with poor prognosis^17^,^19^. Hence, targeting COX-2 activity through nonsteroidal anti-inflammatory drugs (NSAIDs) has been proposed as a potentially promising strategy in the prevention and treatment of cancer^20^. In this context, NSAIDs have been widely employed over the last decades in pre-clinical and clinical trials as COX inhibitors, facilitating anti-tumoral host immune responses (mainly through the reduction of the M2-like polarization of TAMs and the infiltration of regulatory cells), and decreasing the incidence and mortality in several types of cancer^21^,^22^.

Even though COX-2 expression is stimulated by oncogenes, tumor promoters, growth factors and cytokines^23^,^24^, several reports point to an important role of microenvironmental factors, such as tumor hypoxia, in the up-regulation of COX-2 expression and the resultant tumor progression^25^,^26^. Previous *in vitro* studies have shown that COX-2 is up-regulated in endothelial and tumor cells in response to IH to levels that exceed even those observed in cells exposed to continuous hypoxia^27^. However, the IH cyclical pattern applied in the *in vitro* study clearly differs from the IH patterns that are characteristic of OSA, since the former occurs as a consequence of the irregular blood flow caused by aberrant non-periodic occlusions in vessels during tumor growth (lasting from many minutes to hours), while the latter is associated with recurrent apneas (lasting only seconds to minutes). Rodent studies focused on OSA have also reported a causal role of COX-2 in the microglial-specific neuroinflammation and the adverse neurocognitive consequences in response to IH^28^,^29^. Therefore, we used celecoxib (Ce), a well-characterized and specific COX-2 inhibitor, to investigate whether the COX-2/PGE_2_ signaling pathway participates in the IH-induced exacerbation of malignant processes using a mouse model of OSA bearing Lewis lung carcinoma (LLC1) tumors. Furthermore, we also assessed which cell type, namely macrophages or tumor cells, provides the major source of PGE_2_ in response to IH using a well-established *in vitro* model.

## Methods

### Cells and reagents

Mouse Lewis lung carcinoma cells (LLC1) and mouse macrophages (RAW 264.7) (American Type Culture Collection, Manassas, VA) were routinely grown as described in the online Supplementary Material. Celecoxib (Ce) preparation and administration, as well as antibodies for fluorescence-activated cell sorting (FACS) are also detailed in this section.

### Animals, *in vivo* IH exposure and drug treatment

Forty C57BL/6 J 10-week old male mice, obtained from Charles River Laboratories (Saint Germain sur L’Arbresle, France), were maintained in a 12-h light/dark cycle and fed with a regular diet chow and tap water *ad libitum*. All experimental procedures were approved by the Ethical Committee for Animal Research of the University of Barcelona (67/15), following relevant guidelines on the care and use of animals and were authorized by the Generalitat de Catalunya Government (8473).

Mice were randomly exposed to either IH (n = 20) or normoxia (N) (n = 20) using an experimental setting previously described^8^. The IH pattern consisted of cycles of 40 seconds of 21% FiO_2_ followed by 20 seconds 5% FiO_2_ for 6 h/day, 6 days/week. This pattern reproduces the oscillations in the arterial oxygen saturation (95.4% to 62.3%) observed in moderate to severe OSA patients^30^. The control group was identically instrumented, but subjected to N (21% FiO_2_). After one week of exposures to N or IH, ten mice from each group were treated daily with 75 mg/kg body weight of Ce or vehicle (14% DMSO in sesame oil) via oral gavage (Fine Science Tools GmbH, Heidelberg, Germany), and the drugs were administered one hour prior to initiation of IH or N exposures. Therefore, four groups were performed: animals subjected to N or IH and treated with vehicle or Ce (N, N+Ce, IH, IH+Ce, n = 10 each) (Fig. S1). The daily Ce dose employed was below the maximum tolerated for mice (250 mg/kg) and similar to several other studies on the effects of Ce on cancer^31^,^32^.

### Lung tumor induction and assessment of tumor malignancy

Prior to the inoculation of tumor cells in mice, the capacity of LLC1 to respond to PGE_2_ (Cayman Chemicals) was examined *in vitro*. To that end, LLC1 cells were seeded in triplicate on 24-well plates at a density of 65,000 cells/well and treated with 10^−6^ mM PGE_2_. The concentration of PGE_2_ chosen for this assay was similar to that measured in the plasma of different murine cancer models^33^,^34^. Cells were maintained for 40 h at 20% O_2_, 5% CO_2_, 100% humidity and 37°C. At the end-point of the experiments, cells were harvested, stained with trypan blue (Sigma-Aldrich, St. Louis, MO) and live cells were counted in a Neubauer chamber using an optical inverted microscope.

After two weeks of pre-exposures to IH or N, animals were subcutaneously injected with 10^5^ LLC1 cells suspended in 0.2 ml of 1x phosphate buffered saline (PBS) in the right flank. Once tumors became palpable, tumor volume was estimated as described before^13^. All mice were euthanized by exsanguination followed by cervical dislocation at day 21 post-injection. Tumors were excised, weighted and the disruption of capsule with presence of invasion toward the skeletal muscle was visually assessed by two blinded investigators. Samples were prepared for FACS, TAMs isolation and western blot (WB). Lung lobes were extracted, fixed in Histofix (Panreac) and paraffin-embedded. Blocks were cut into 4 μm sections, stained with hematoxylin/eosin and blindly examined under light microscopy to assess metastatic potential by one of the investigators who was unaware of the treatment group. Total number of LLC1 metastases, relative metastatic area and mean size of each metastasis per group were calculated. Histological sections were also performed in some tumor samples and examined under microscopy to confirm findings regarding tumor invasion. COX-2 protein levels in the tumor were examined by WB (see online Supplementary Material for detailed protocol).

### Assessment of Tregs, MDSCs and TAMs populations by flow cytometry

Tumors were minced and digested in a 1 mg/mL collagenase type IA (Gibco, Gaithersburg, MD) solution for 1 h at 37 °C. Cells were filtered through a 100 μm nylon mesh cell strainer (BD Falcon, Bedford, MA) and stained with fluorophore-conjugated antibodies for the identification and quantitative analysis of Tregs, TAMs and MDSCs populations by FACS as previously described^13^. Labelled cells were analyzed on a FACS Canto II cytometer using FACS Diva 5.5 software (BD Biosciences, San Jose, CA) and analyzed by FlowJo software (Tree Star, San Carlos, CA). The subpopulation of Tregs was identified by FoxP3 and CD45+, CD3+, CD4+, CD8-, CD25+ cell surface markers, while MDSCs were recognized by CD45+, CD11b+ and Gr-1+ markers, and TAMs as CD45+, CD11b+ and F4/80+. Polarization of TAMs towards M2 or M1 phenotype was assessed by measuring CD206 or CD86 surface markers, respectively. In addition, the M2/M1 ratio was computed from the quotient between the mean fluorescence intensity (MFI) values for CD206 and CD86 markers. Isotype controls were employed to establish background fluorescence. Cell populations were measured as cells per gram of tumor and total numbers of TAMs, Tregs and MDSCs recruited within the tumor.

### Determination of M1/M2 TAMs phenotype by real-time polymerase chain reaction

Gene expression analyses of M1 (INF-γ, interferon gamma) and M2 markers (MRC1, mannose receptor C type 1; IL-10, interleukin 10) by real-time polymerase chain reaction (qPCR) were performed in isolated TAMs to further assess their polarization. Firstly, TAMs were purified from digested tumors by CD11b-PE magnetic labelling (EasySep^TM^Mouse CD11b Positive Selection Kit, StemCell Technologies, Vancouver, BC) following the manufacturer’s protocol. Then, total RNA from isolated TAMs was extracted using the RNeasy kit (Qiagen, Hilden, Germany) and 0.5 μg were used to synthesize cDNA by reverse transcription polymerase chain reaction, according to manufacturers’ instructions. qPCR was performed using the Taqman Gene Expression Master Mix (ThermoFisher Scientific, Waltham, MA) in a StepOnePlus thermocycler (ThermoFisher Scientific, Waltham, MA). Relative gene expression normalized to eukaryotic 18 S rRNA endogenous control was calculated using the 2^−∆∆CT^ method to quantify fold change in gene expression of IFN-γ, IL-10 and Mcr1 compared to baseline expression (N group). Taqman Gene Expression Assays were purchased from ThermoFisher Scientific. PTGS2 (prostaglandin-endoperoxide synthase 2) relative gene expression was also assessed in TAMs from N and IH groups to complement data regarding COX-2 protein expression in tumors.

### Co-culture of tumor associated macrophages with LLC1 cells *in vitro*

Isolated TAMs were co-cultured with LLC1 naïve cells (4:1 ratio) in full media for three days^13^. At the end point, cells were harvested, labeled with CD45-FITC and both cell types were identified (TAMs as CD45+ cells and LLC1 as CD45- cells) and counted by FACS to determine LLC1 proliferation.

### Assessment of PTGS2 gene expression and PGE_2_ production by LLC1 and RAW 264.7 cells exposed to IH *in vitro*

LCC1 and RAW 264.7 cell exposures to N (20% O_2_) or IH (30 s 1% O_2_–30 s 20% O_2_) were performed using a custom-made system adapted from a chip previously described^35^. The chip employed herein is composed by two PDMS layers designated as gas and culture chambers containing six PDMS wells of 12 mm diameter each. Both chambers are separated by a 20 μm thick PDMS membrane (Fig. S2a,b). Wells of the gas chamber are connected in series allowing circulation of gases from a gas source providing a constant mixture of gases (N) or oscillations in gas composition (IH) to the six independent cell cultures adhered on top of the membrane (culture chamber). Oxygen measurements performed on top of membrane (at the cell culture level) using a fiber-optic oxygen sensor (FireSting O_2_, Pyro Science, Aachen, Germany) showed that, in agreement with our previous study, the system displays a fast equilibration time (~6 s) due to fast oxygen diffusion through the thin membrane (Fig. S2c). Therefore, oxygen desaturation patterns experienced by OSA patients can be replicated at the cell culture level or in living animal tissues using the *in vitro* and *in vivo* models described herein, respectively.

Prior to experiments, the viability of LLC1 and RAW 264.7 cells at different concentrations of Ce was tested such as to select the optimal concentration and avoid toxicity-induced cell death (see Supplementary Fig. S3). LLC1 and RAW 264.7 single cultures treated with Ce (10 μM) or vehicle (0.05% DMSO) were exposed to either IH (30 s 1% O_2_–30 s 20% O_2_) or N (20% O_2_) for 24 h (Fig. S2d). Then, supernatants were collected, centrifuged at 300 g for 5 min to discard possible cells in suspension and total levels of PGE_2_ (pg) in cell media supernatants were determined using mouse PGE_2_ ELISA kit (Cayman Chemical, Tallinn, Estonia), according to the manufacturer’s protocol. RAW264.7 and LLC1 cells were harvested and PTGS2 relative gene expression was assessed as described above for the *in vivo* study.

### Statistical analysis

Data are reported as mean ± standard error (SE). Shapiro-Wilk tests were performed to ensure normal distribution of samples prior to analysis. Data from N, N+Ce, IH, IH+Ce groups were compared using two-way analysis of variance (ANOVA). Gas and drug exposures constituted the variables. Student-Newman-Keuls post hoc test was used for multiple comparisons. T-test analyses were used to compare LLC1 *in vitro* proliferation in presence or absence of PGE2, COX-2 protein levels in tumors and PTGS2 gene expression in TAMs from N and IH groups. Differences were considered significant for p < 0.05. Statistical analyses were performed with SigmaPlot (Systat Software, San Jose, CA).

## Results

### Celecoxib inhibits IH-induced tumor malignancy changes

**Figure 1:**
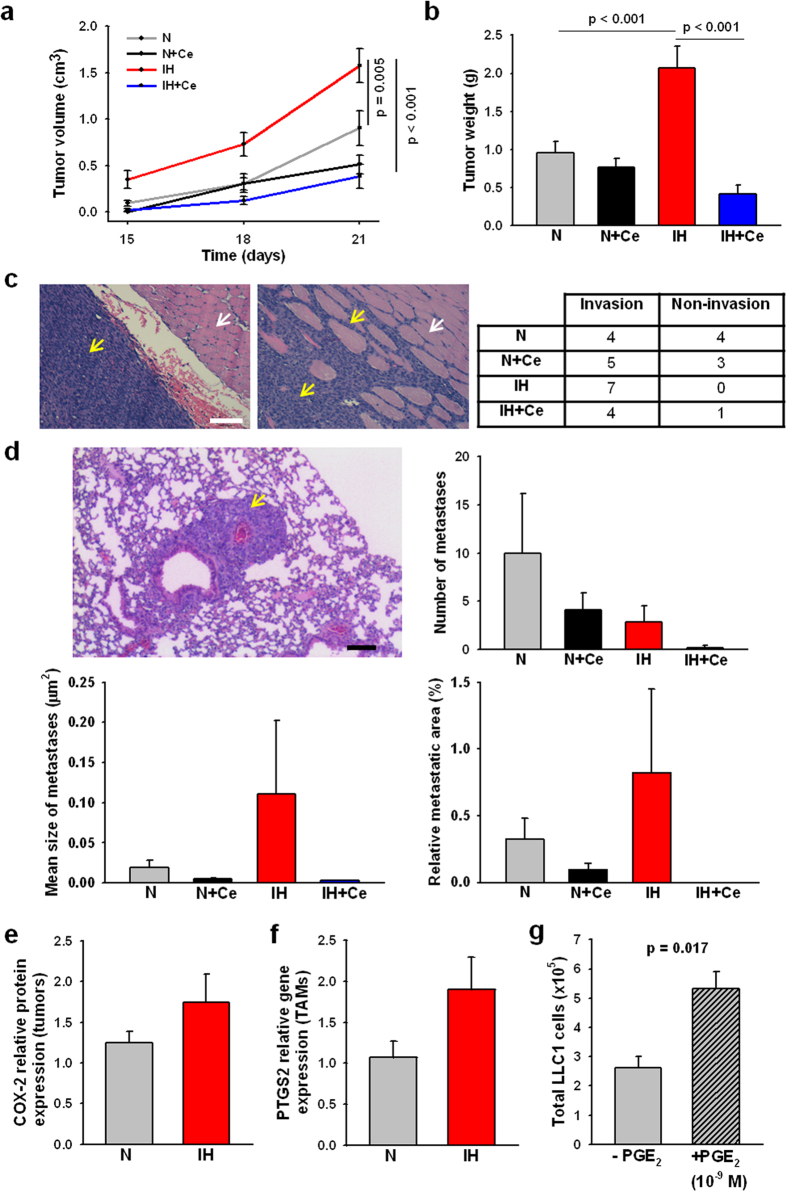
Tumor growth and invasion in celecoxib (Ce) or vehicle-treated mice exposed to normoxia (N) or intermittent hypoxia (IH). (**a**) Periodic tumor volume measurements revealed an accelerated tumor growth in IH-treated mice compared to N, which was inhibited by Ce administration. (**b**) Weight average of tumors from mice exposed to IH experienced ~2-fold increase respect to N. Under IH conditions, daily administration of Ce promoted a markedly reduction in tumor weight. (**c**) Representative histological sections of the tumor and the adjacent muscle presenting non-invasion (left) or invasion (right). LLC1 cells are predominantly stained by hematoxylin (dark blue, yellow arrows) whereas myocytes are more intensely stained with eosin (pink, white arrows). Scale bar = 100 μm. The number of mice presenting invasion was higher in IH vs N exposed mice. (**d**) Number of lung metastases, mean size, and relative metastatic area do not exhibit significant variations among the different treatments due to a high intra-group variability. Representative image of a metastasis (yellow arrow) in the lung parenchyma. Scale bar = 100 μm. Tumors (**e**) and purified TAMs (**f**) from IH-exposed mice showed a trend to an increased COX-2 protein and PTGS2 gene relative expression, respectively. (**g**) LLC1 proliferation in response to exogenous PGE_2_ *in vitro*. Data are presented as mean ± SE.

IH exposures promoted accelerated tumor growth from tumor weight and tumor volume measurements (2.07 g ± 0.28 and 1.57 cm^3^ ± 0.18, respectively) when compared to normoxic mice (0.95 g ± 0.15, p < 0.001; 0.90 cm^3^ ± 0.19 p = 0.005) (Fig. 1a,b). Similarly, the number of mice presenting evidence of local tumor invasion in surrounding tissues was higher in mice exposed to IH (Fig. 1c). No statistically significant differences emerged in the total number of LLC1 metastases, relative metastatic area, and mean size of metastases among the different groups (Fig. 1d). While the number of metastases slightly increased in mice exposed to N, the mean size of the metastases was lower than the observed in the IH groups. Nevertheless, the large variability among samples within the same treatment group hindered the statistical analyses. Tumors from IH-exposed mice presented a trend to an increased COX-2 protein expression in comparison to N (p = 0.284) (Fig. 1e). Similarly, isolated TAMs also exhibited a trend to increased expression of the PTGS2 gene (p = 0.106) (Fig. 1f). Daily administration of Ce markedly decreased IH-induced tumor growth (tumor weight = 0.42 g ± 0.12; tumor volume = 0.38 cm^3^ ± 0.13, p < 0.001) (Fig. 1a,b). Application of PGE_2_ to LLC1 cells *in vitro* elicited a 2-fold increase in proliferation (p = 0.017), confirming that this tumor model functionally responds to PGE_2_ (Fig. 1g).

### Celecoxib suppresses the IH-induced alterations in the host immune response

**Figure 2:**
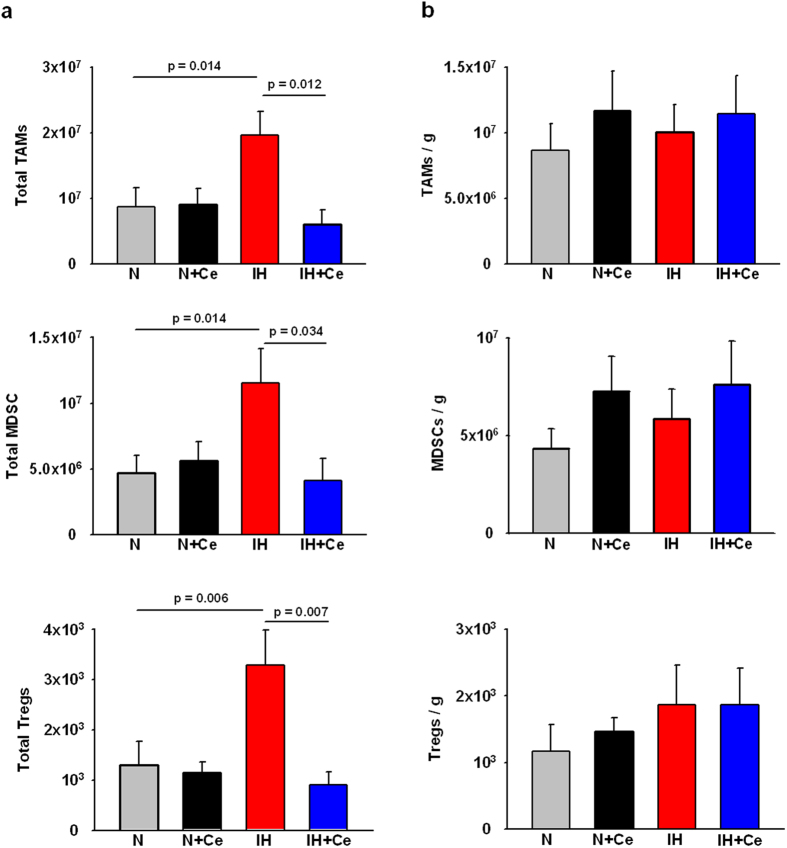
Tumor associated macrophages (TAMs), myeloid-derived suppressor cells (MDSCs) and regulatory T cell lymphocytes (Tregs) within the tumor. (**a**) Application of IH resulted in an enhanced tumor weight and hence increased total number of TAMs, MDSCs and Tregs populations within the tumor, while Ce administration prevented tumor growth and therefore reduced cell numbers. b) No significant differences in cell population density (cells/g of tumor) of TAMs, MDSCs and Tregs were observed amongst different groups. Data are presented as mean ± SE.

In absence of Ce treatment, a 2-fold increase (p = 0.014) in TAMs population and 2.5-fold increases in MDSCs (p = 0.014) and Tregs (p = 0.006) counts emerged in tumors from mice exposed to IH when compared to the N group (Fig. 2a). Conversely, in IH-exposed animals receiving Ce, TAMs, Tregs and MDSCs counts were restored to N basal levels. When normalizing for tumor weight (Fig. 2b), the number of TAMs, MDSCs and Tregs remained similar among the different conditions. See Fig. S4 for representative flow cytometry dot plots of gated populations.

**Figure 3:**
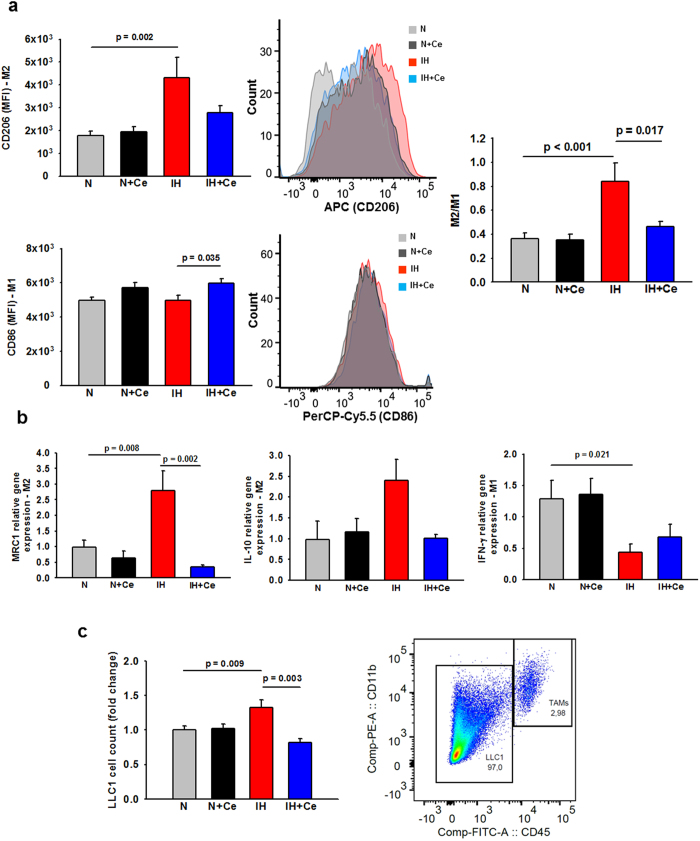
Polarization of tumor-associated macrophages (TAMs) and their effects on LLC1 cell proliferation. (**a**) Assessment of CD206 (M2 phenotype marker, upper) and CD86 (M1 phenotype marker, lower) in TAMs by FACS as well as representative mean fluorescence intensity (MFI) histograms of each marker in N, N+Ce, IH and IH+Ce samples. Up-regulation of M2 marker was observed in TAMs from mice that underwent IH treatment, whereas Ce resulted effective avoiding M2-like polarization. M2/M1 ratio calculated from the quotient between CD206 and CD86 MFIs increased consistently in IH-exposed animals, and was prevented in IH-treated mice that received Ce (center). (**c**) Proliferation of LLC1 cells co-cultured with TAMs isolated from mice. LLC1 cells increased their proliferation when co-cultured with TAMs obtained from IH-exposed mice, whereas IH+Ce TAMs significantly reduced LLC1 cell proliferation respect to the IH-treated group. Representative dot plot depicting the gating performed to identify CD45+, CD11b+ (TAMs) and CD45−, CD11b− (LLC1) populations during FACS analysis. Data are presented as mean ± SE, MFI = Mean fluorescence intensity (arbitrary units).

Most importantly, IH exposures induced a polarity shift in TAMs from an anti-tumoral phenotype (M1) towards a tumor-promoting phenotype (M2), as reflected by a ~2-fold increase in M2/M1 ratios when compared to mice exposed to N (p < 0.001) (Fig. 3a). Accordingly, qPCR analyses carried out on isolated TAMs revealed that IH treatment downregulated the expression of the M1 marker INF-γ (p = 0.021) while the expression of the M2 markers MCR1 (p = 0.008) and IL-10 (p = 0.083) was up-regulated, in comparison to N (Fig. 3b). On the other hand, the M2/M1 ratios in Ce-treated mice exposed to IH as well as the expression of genes associated with M1 and M2 phenotypes were restored to levels similar to those found among tumors in normoxic mice. No changes in TAMs polarization were observed between N and N+Ce groups.

Co-culture of naïve LLC1 with TAMs from tumors of IH-exposed mice induced increased proliferation relative to N-exposed TAMs (Fig. 3c, p = 0.009). However, when LLC1 cells were co-cultured with TAMs from IH+Ce-exposed mice, cell numbers were significantly decreased to levels slightly below those of LLC1 co-cultured with N TAMs (p = 0.003).

### Celecoxib inhibits the increased PGE_2_ production by LLC1 and RAW 264.7 cells in response to IH *in vitro*

**Figure 4:**
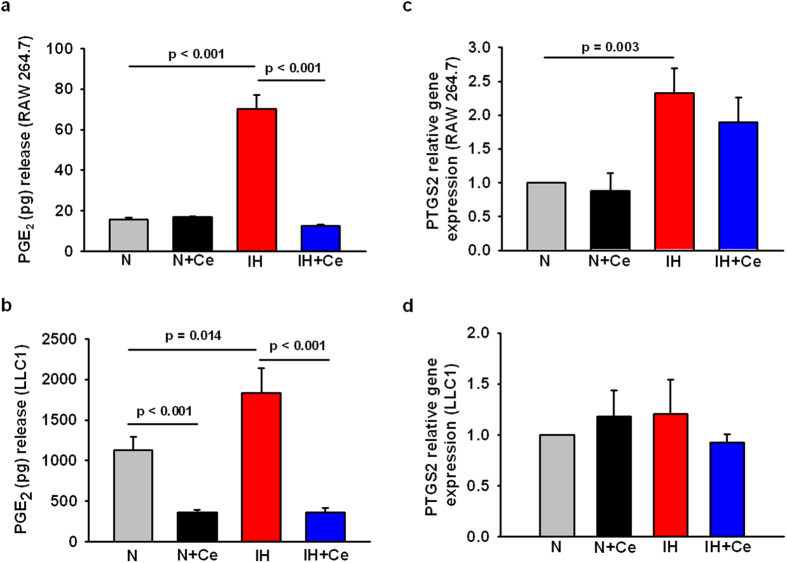
Alterations in the COX-2/PGE_2_ signaling pathway in by RAW 264.7 and LLC1 cells exposed to IH and/or Ce treatment *in vitro*. PGE_2_ secretion by RAW 264.7 macrophages (**a**) and LLC1 cells (**b**) exposed to IH increased significantly in comparison to N, while Ce administration resulted in a reduction of PGE_2_ production in both cell types near to N levels (RAW 264.7 cells) or even below (LLC1 cells). PTGS2 relative gene expression was up-regulated in RAW264.7 cells in response to IH (**c**) while no changes were detected in the case of PTGS2 by LLC1 cells between different treatments (**d**). Data are presented as mean ± SE.

RAW 264.7 macrophages and LLC1 cells exposed to IH for 24 h exhibited ~4.5-fold and ~1.5-fold increases in PGE_2_ production, respectively (Fig. 4a, n = 6 p < 0.001; Fig. 4b, n = 5, p = 0.014), when compared to normoxic conditions. Similarly, IH induced an up-regulation of PTGS2 expression in macrophages (p = 0.003), while no changes were observed in the PTGS2 gene expression in tumor cells (Fig. 4c,d). The addition of 10 μM Ce to the cell media significantly reduced PGE_2_ secretion to levels observed under normoxia (RAW 264.7) or even below (LLC1 cells) (p < 0.001), while PTGS2 gene expression did not change in response to Ce treatment.

## Discussion

In this study, we found that the IH-induced enhancements of tumor malignancy are mediated, at least in part by the COX-2/PGE_2_ signaling pathway. The application of the COX-2 specific inhibitor Ce suppressed IH-induced alterations in the host immune response within the tumor microenvironment, leading to a marked reduction in tumor growth. Furthermore, LLC1 cells increased their proliferative rates in response to exogenous PGE_2_ at concentrations previously measured in several types of cancer^33^,^34^. *In vitro* exposure of macrophages and LLC1 cells to IH resulted in the activation of the COX-2/PGE_2_ pathway, suggesting that these cells are likely sources of PGE_2_ in the tumor microenvironment and also can further increase in response to IH.

Recent clinical and translational studies have suggested a link between OSA and increased cancer incidence, aggressiveness and mortality^5^,^6^,^7^,^8^,^9^. Animal studies have further revealed that IH exposures that mimic OSA-induced oxygenation patterns during sleep lead to accelerated tumor growth, invasiveness and metastasis in certain types of tumors such as lung carcinoma^13^ and melanoma^9^. In the present study, LLC1 tumors had similar responses to IH as those reported in previous studies with other types of cancer cells^8^,^9^,^13^. Concretely, IH led to increased tumor growth and invasiveness in LLC1 tumors. In contrast to previous findings, no changes in tumor metastases were observed, a finding that may be explained by the highly aggressive metastatic behavior of these cells even in control conditions^32^. PTGS2 gene and COX-2 protein expression showed a trend toward increases in purified TAMs and in tumors isolated from IH-exposed mice in comparison to normoxia, respectively. Given that the biological material available to perform WB and qPCR analyses was limited owing to the reduced size of tumors (especially in Ce groups) and that samples were mostly used to perform FACS analyses and TAMs co-cultures with LLC1, it becomes apparent that larger sample sizes would be desirable to draw a more definitive conclusion. Regarding the immune alterations promoted by IH, our results are closely concordant with previous data on other tumors^13^,^14^. FACS analysis revealed alterations in TAMs polarity from the classically activated anti-tumoral M1 phenotype towards an alternatively activated pro-tumoral M2 phenotype. Accordingly, qPCR analyses further confirmed the enhanced expression of MRC1 and IL-10 genes (M2 markers) and the decreased expression of IFN-γ (M1 marker) in TAMs isolated from IH-exposed mice. In fact, the polarity switch of TAMs is currently considered a key step in the acceleration of cancer aggressiveness, and has been directly associated with a poor prognosis in several types of tumors^36^,^37^. Despite the fact that IH-exposed tumors had increased TAMs, MDSCs and Tregs counts, the number of cells per gram of tumor remained similar among groups, suggesting that the increased tumor aggressiveness observed in animals treated with IH is most likely explained by a change in TAMs phenotype rather than ascribable to an increased infiltration of other immunosuppressive populations. Other studies observed an accumulation of MDSCs and Tregs which was associated with increased malignant properties, since both populations impair tumor immunosurveillance by limiting the infiltration and functions of cytotoxic populations^38^, while also promoting the M2-like polarization of TAMs^39^.

There is increasing evidence suggesting that biochemical signals participating in the cross-talk between tumor cells and tumor stromal cells are essential for cancer escape (i.e. transcription factors, growth factors and some cytokines)^40^. In addition, biophysical cues of the tumor microenvironment (i.e. hypoxia^41^ or extracellular matrix components which can modulate its stiffness^42^) have also been implicated. PGE_2_, a downstream product of COX-2 enzymatic activity, has been identified as a major orchestrator, coordinating the pro-angiogenic and immunosuppressive programs that facilitate tumor growth and contribute to cancer progression^16^,^18^. Regarding immunosuppression, a large body of evidence indicates that PGE_2_ plays an important role in the initiation and maintenance of M2 polarized TAMs, as well in promoting the suppression of M1 polarity^22^,^43^. In addition, PGE_2_ downregulates anti-tumoral Th1 responses while promoting pro-tumoral Th2 responses of T helper cells^44^, limits the proliferation and functionality of cytotoxic T-lymphocytes and natural killer (NK) cells^16^,^45^, induces the recruitment of Tregs and MDSCs populations^16^, and inhibits the differentiation of dendritic cells^46^. Moreover, PGE_2_ also increases the proliferation, survival, migration and invasion of tumor cells through its binding to their specific receptors^47^. In fact, high levels of PGE_2_ in the tumor resulting from the aberrant activation of COX-2 are commonly considered as indicators of a poorer cancer prognosis in different types of malignancies such as melanoma, breast, colon, lung, or gastric cancer^17^. In this study, LLC1 cells exposed *in vitro* to PGE_2_ augmented their proliferative rate revealing their susceptibility to respond to this inflammatory mediator likely through its binding to the EP4 receptor^48^ (Fig. 1g). Although COX-2 up-regulation in cancer cells has been classically linked to the activation of oncogenes or transcription factors, it has been also detected in tumor cells and other cells of tumor stroma in response to hypoxia, via stabilization of hypoxia inducible factor 1 (HIF-1) and nuclear factor kappa-light-chain-enhancer of activated B cells (NF-κB)^25^,^26^,^49^. In fact, hypoxic regions of the tumor positively correlate with increased COX-2 expression, accumulation of M2-like polarized macrophages and other immunosuppressive cells^41^.

In the context of IH, a previous study showed the potential involvement of COX-2 in the transcriptomic adaptation of endothelial and tumor cells to low frequency cyclic hypoxia^27^. Similarly, studies in the field of OSA, also reported the participation of COX-2 in the pathogenesis of OSA-related morbidities^28^. Thus, the link between COX-2 and IH is not only biologically plausible, but further appears to underlie components of the enhanced aggressive biological properties of cancer in the context of OSA. To characterize the potential implications of COX-2/PGE_2_ signaling pathway in the IH-enhanced tumor growth and aggressiveness in the mouse model of OSA bearing LLC1 tumors, we employed Ce, a specific inhibitor of COX-2. Experimental studies and clinical trials have been carried out over the last decades to assess the potential therapeutic role of Ce and other COX-2 inhibitors in the prevention and treatment of different types of cancer^20^,^21^. Although most clinical trials point to a better prognosis in patients receiving Ce treatment in addition to chemo-, radio- or chemoradiotherapy, there is still a lack of consensus and certainly some degree of controversy about the therapeutic role of this compound, probably due to the presence of multiple other confounding factors affecting cancer outcomes^50^,^51^. Notwithstanding, the effectiveness of Ce in delaying tumor growth and improving cancer prognosis has been shown in several types of cancer both *in vivo* and *in vitro*, when experimental conditions were tightly controlled^22^,^32^. In the present study, the effect of Ce on tumor growth was evident in IH-exposed animals, and induced 5-fold reductions in tumor weight and volume. In addition, Ce reduced the number of animals presenting tumor invasion to adjacent muscle. These improvements in cancer prognosis could be partially explained by the recovery of the anti-tumoral responses of the immune system as reflected by increases in M1 polarized TAMs and reductions in the M2-like polarization of TAMs through the specific inhibition of COX-2 by Ce, as determined by FACS, qPCR and ELISA analyses. Similar findings were previously observed in murine models of cancer that were unrelated to OSA or IH^22^,^52^. Some studies have also documented an increase in the infiltration of cytotoxic T-lymphocytes and NK cells^53^. Although we did not assess the effects of IH and/or Ce on the recovery of such immune populations, we would anticipate similar findings based on the reduction of PGE_2_ production by Ce.

The *in vitro* experiments carried out with isolated TAMs from tumors confirmed that the phenotypic changes have implications in LLC1 proliferation in co-culture. Similar to our previous findings^13^, LLC1 naïve cells co-cultured with TAMs from mice exposed to IH showed increased proliferation over those co-cultured with N-exposed TAMs. When co-cultured with TAMs from IH-exposed animals treated with Ce, LLC1 cells significantly reduced their proliferation. Moreover, LLC1 single cultures showed higher proliferative capacity when exogenous PGE_2_ at a concentration similar to that found in plasma from different mouse models of cancers^33^,^34^ was added to the media. These findings confirm that PGE_2_ is able to directly induce tumor cell growth in our model, most likely through binding to specific receptors in LLC1 cells.

Macrophages and tumor cells (RAW 264.7 and LLC1 cell lines, respectively) were single cultured and exposed to IH *in vitro* in order to identify which cells is the major source of PGE_2_ in the tumor in response to IH. qPCR and ELISA analyses revealed that IH induced significant increases in PTGS2 gene expression and PGE_2_ production by macrophages. In tumor cells, PGE_2_ production was also increased in response to IH; however, no changes on PTGS2 gene expression were detected. This finding could be explained by the increased expression of PTGS2 on tumor cells even in basal conditions^23^,^24^, with IH most likely being associated with modulation of expression at the protein level. Addition of Ce to the media inhibited PGE_2_ production, supporting the efficiency of this drug in delaying tumor malignancy by reducing PGE_2_ secretion by TAMs and cancer cells and also their positive-feedback, hence supporting the findings *in vivo*^43^. Taken together, these results suggest that despite the fact that macrophages are more prone to exhibit alterations in the COX-2/PGE_2_ pathway in response to IH, both cell types (macrophages and LLC1 cells) are relevant sources of PGE_2_ in our murine model of OSA.

In conclusion, the increased tumor malignant properties associated with IH appear to be mediated, at least in part, by the up-regulation of the COX-2/PGE_2_ pathway in tumor cells and macrophages. Our findings provide further support to the concept that NSAIDS, and more concretely Ce, could provide effective benefits in the treatment of tumors whose proliferative properties and vascularity patterning make them more susceptible to regional hypoxic cycles, as well as in the context of cancer patients suffering from OSA.

## Additional Information

**How to cite this article**: Campillo, N. *et al*. Role of Cyclooxygenase-2 on Intermittent Hypoxia-Induced Lung Tumor Malignancy in a Mouse Model of Sleep Apnea. *Sci. Rep.* **7**, 44693; doi: 10.1038/srep44693 (2017).

**Publisher's note:** Springer Nature remains neutral with regard to jurisdictional claims in published maps and institutional affiliations.

## Supplementary Material

Supplementary Material
