# Supplementary material for: Role of Cyclooxygenase-2 on Intermittent Hypoxia-Induced Lung Tumor Malignancy in a Mouse Model of Sleep Apnea
Source: Sci Rep. 2017 Mar 16;7:44693. doi: 10.1038/srep44693 (PMC5353645; doi:10.1038/srep44693)
Supplement: Supplementary Material [file srep44693-s1.doc]

**ONLINE SUPPLEMENTARY MATERIAL**

**ROLE OF CYCLOOXYGENASE-2 ON INTERMITTENT HYPOXIA-INDUCED LUNG TUMOR MALIGNANCY IN A MOUSE MODEL OF SLEEP APNEA**

Noelia Campillo, Marta Torres, Antoni Vilaseca, Paula Naomi Nonaka, David Gozal, Jordi Roca-Ferrer, César Picado, Josep Maria Montserrat, Ramon Farré, Daniel Navajas, Isaac Almendros

**Cells and reagents**

Mouse Lewis lung carcinoma cells (LLC1) and mouse macrophages (RAW 264.7) (American Type Culture Collection, Manassas, VA) were routinely grown in high glucose Dulbecco's modified Eagle's medium (DMEM) supplemented with 10% fetal bovine serum (FBS) (Gibco), and an antibiotic/antimycotic solution at final concentrations of 100 U/ml penicillin, 100 µg/ml streptomycin and 0.250 µg/ml amphotericin B (Sigma-Aldrich). Cells were cultured in 75 cm^2^ tissue culture flasks (Techno Plastic Products, Trasadingen, Switzerland) and maintained in a standard humidified incubator at 20% O_2_, 5% CO_2_ and 37ºC. For *in vitro* experiments, Celecoxib (Ce) was dissolved in DMSO (both from Sigma-Aldrich) to a final concentration of 20 mM and stored in aliquots at -80ºC until its use. For animal studies, Ce (Cinfa, Navarra, Spain) was prepared daily for oral administration as described before^31^. All antibodies for fluorescence-activated cell sorting (FACS) were purchased from BioLegend (San Diego, CA): Gr-1-PerCP (clone RB6-8C5), CD11b-PB (clone M1/70), CD45-FITC (clone30-F11), F4/80-PE (clone BM8), CD206-APC (clone C08C02), CD86-PerCP/Cy5.5 (clone GL-1), Forkhead box P3 (FoxP3) (clone MF-13), CD25-PerCP/Cy5.5 (clone 3C7), CD3-APC (clone 145-2C11), CD4-APC/Cy7 (clone RM4-5) and CD8-PB (clone 53-6.7).

**Determination of COX-2 protein expression in tumors**

Proteins were extracted from snap-frozen tumor samples using lysis buffer containing 0.5% triton X-100, 5 mM Hepes (Sigma-Aldrich) and protease inhibitors (Roche, Basel, Switzerland), for 10 min at 4⁰C. Samples were sonicated at 40% amplitude during 20 s and centrifuged at 13,000 g for 10 min at 4⁰C. Protein content in supernatants were measured using the Pierce bicinchoninic acid (BCA) protein assay (ThermoFisher Scientific). 100 µg of total protein were separated using 10% mini-PROTEAN TGX^TM^ Precast Gels (Bio-Rad, Barcelona, Spain) and blotted onto activated PVDF membranes (GE Healthcare, Amersham, UK). Membranes were probed with rabbit polyclonal anti-COX-2 (Abcam, Cambridge, UK) and mouse monoclonal anti-β-actin (Sigma-Aldrich) antibodies at 1:400 and 1:20,000 dilutions, respectively. Horseadish peroxidase-conjugated anti-rabbit and anti-mouse antibodies (ThermoFisher Scientific) were allowed to bind to the primary antibodies and detected by chemiluminescence using Luminata^TM^ Forte Western HRP Substrate (Merck MilliPore, Madrid, Spain). Relative quantification of COX-2 expression normalized to β-actin was computed from chemiluminiscence images using ImageJ software (http://imagej.nih.gov/ij/).

**Celecoxib preparation and cell viability assay**

The effect of Ce on cell viability was determined by trypan blue exclusion assay. To that end, RAW 264.7 and LLC1 cells were individually cultured into 24-well plates at densities of 1.5 x 10^4^ and 6.5 x 10^4^ cells per well, respectively. Different concentrations of Ce (0, 1, 10 and 100 µM), or growing concentrations of DMSO (up to 0.5%) as control assays, were added during cell seeding. Assays were performed per duplicate and cells were maintained in a standard cell culture incubator (20% O_2_, 5% CO_2_, 100% humidity and 37⁰C), and were harvested 40 hours later. Cell suspension was diluted (1:1) in trypan blue (Sigma-Aldrich) and cells were counted in a Neubauer chamber using an optical inverted microscope. Cell viability (v) was calculated as the number of viable cells divided by the total number of cells and expressed as percent (v x 100) (Fig. S3).

**SUPPLEMENTARY FIGURES**

**
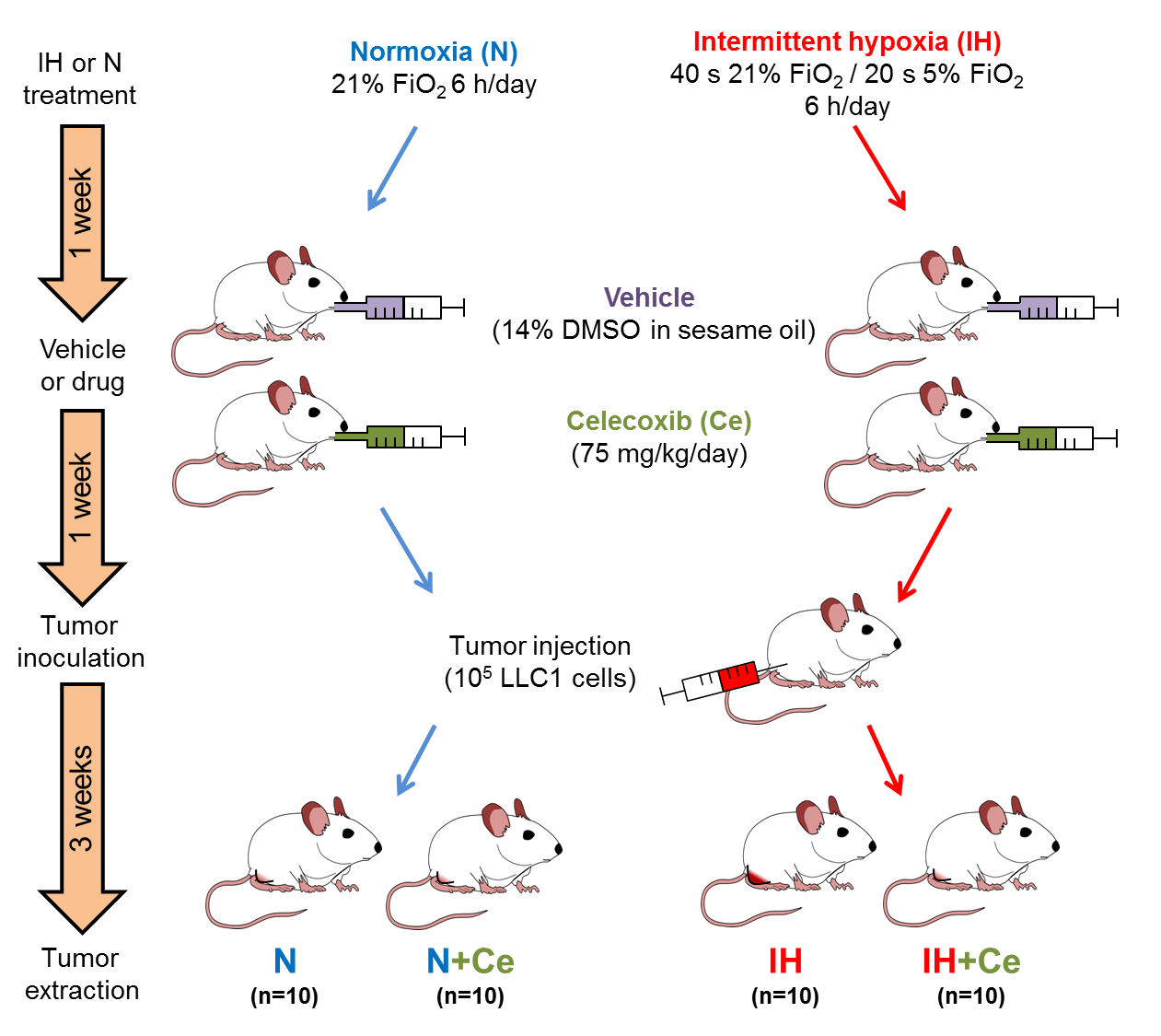
**

**Figure S1.** *In vivo* experimental design. Animals were exposed to either normoxia (N) or intermittent hypoxia (IH). After 1 week, 10 mice from each group (IH or N) were randomly treated daily with celecoxib or vehicle until the end of the experiment. One week later, all animals were injected with 10^5^ lung carcinoma cells diluted in PBS in the right flank. Finally, 3 weeks post-injection, all mice were anesthetized, euthanized and tumors were excised for further analyses.


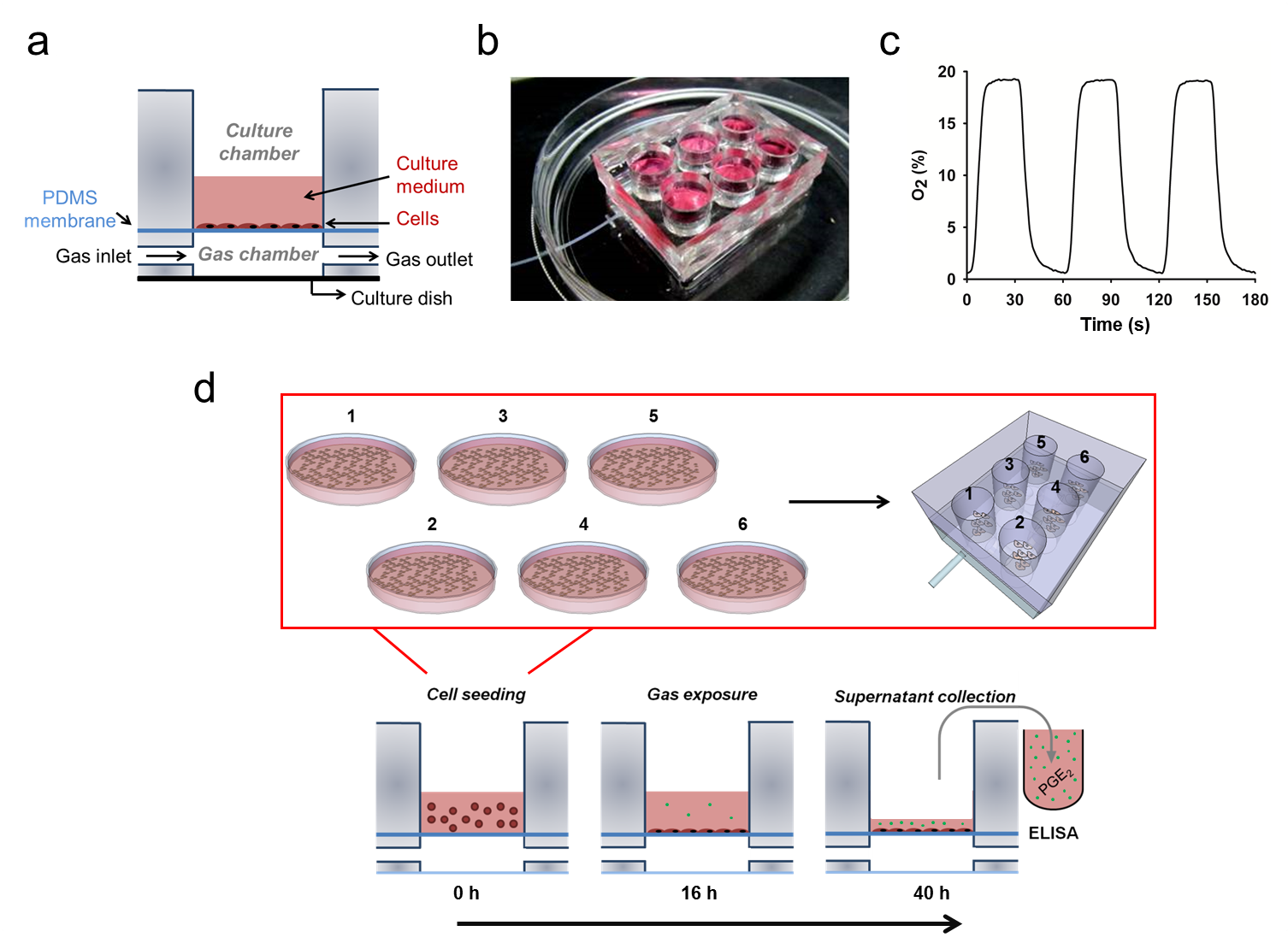


**Figure S2.** Experimental system employed for *in vitro* cell exposures to normoxia (N) and intermittent hypoxia (IH). a) Schematic view of a well and b) the entire chip containing 6 wells. c) Oxygen profiles measured on top of membrane (at the cell culture level) using a fiber-optic oxygen meter when step changes in P_O2_ patterns mimicking OSA (30 s 0% O_2_ – 30 s 20% O_2_) were provided by the gas source. d) Experimental design of *in vitro* experiments for LLC1 and RAW 264.7 cell exposures to N or IH. Cells from six subcultures at the same passage were seeded in the corresponding wells of the chip. Four identical chips were connected to either N or IH in the presence/absence of celecoxib (Ce).

**
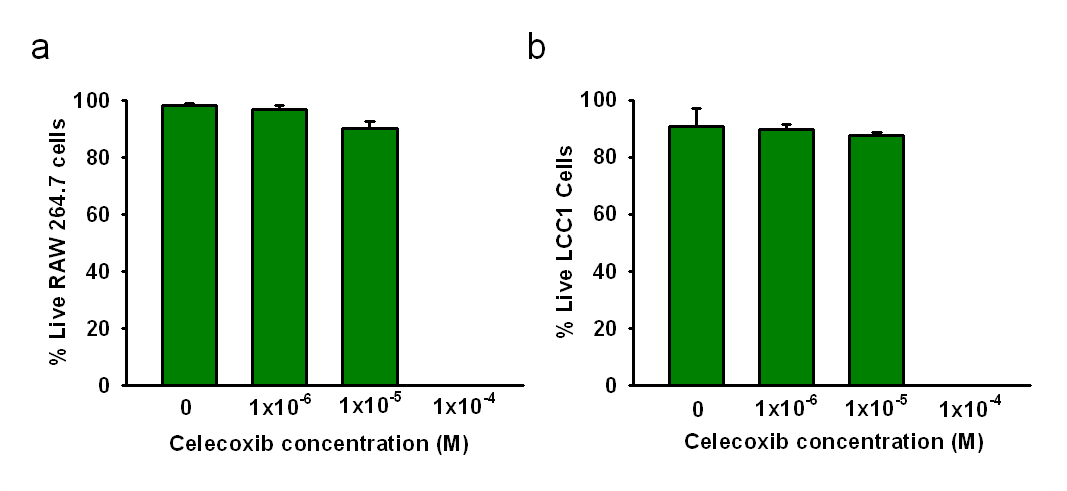
Figure S3**. Effects of Ce on the viability of RAW 264.7 and LLC1 cells. The addition of Ce to the cell media at concentrations below 10 µM had marginal effects on the viability of RAW 264.7 and LLC1 cells, as determined by trypan blue exclusion assay. Ce at a concentration of 100 µM dramatically reduced cell viability in both cell types. A concentration of 10 µM Ce was employed for *in vitro* experiments.


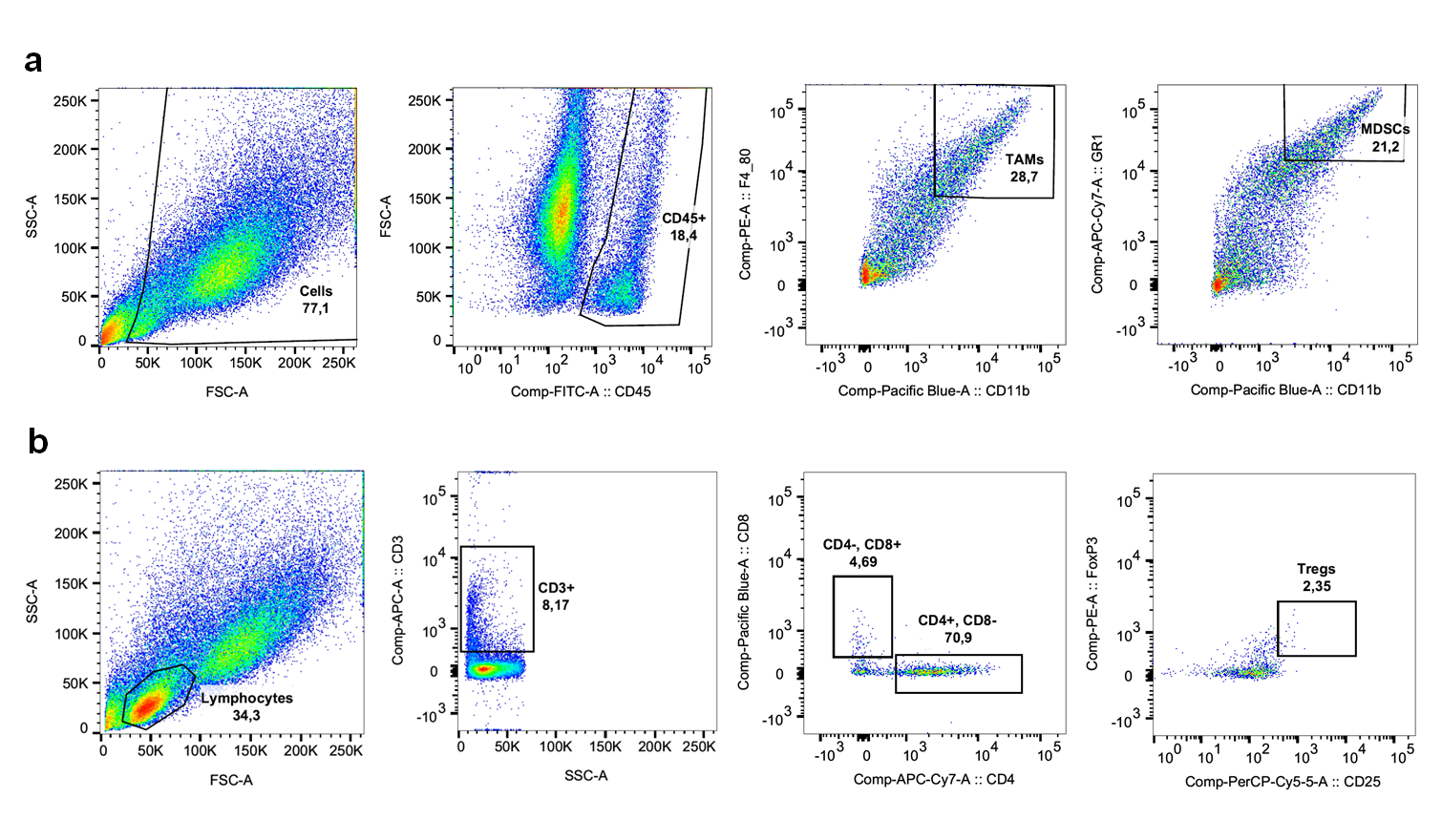
**Figure S4.** Representative dot plots indicating the gating strategy to identify tumor-associated macrophages (TAMs) and myeloid derived suppressor cells (MDSCs) (a) or regulatory T lymphocytes (Tregs) (b) subpopulations. TAMs were identified by CD45+, CD11b+ and F4/80+ cell surface markers, while MDSCs were recognized by CD45+ CD11b+ and Gr-1+ markers, and Tregs by CD45+, CD3+, CD4+, CD8-, CD25+ cell surface markers and FoxP3.
